# Effects of Ving Tsun sticking-hand training on lower limb sensorimotor performance among community-dwelling middle-aged and older adults: a randomized controlled trial

**DOI:** 10.1186/s13063-023-07133-2

**Published:** 2023-02-25

**Authors:** Shirley S. M. Fong, Louisa M. Y. Chung, Timothy T. T. Yam, Joanne W. Y. Chung, Young-Hyeon Bae, Yang Gao, Jessie S. M. Chan, Hsing-Kuo Wang

**Affiliations:** 1 Department of Health and Physical Education, The Education University of Hong Kong, Tai Po, Hong Kong; 2 Hong Kong Martial Arts Institute, Kowloon, Hong Kong; 3 Department of Physiotherapy, School of Nursing and Health Studies, The Hong Kong Metropolitan University, Kowloon, Hong Kong; 4 School of Nursing and Health Studies, The Hong Kong Metropolitan University, Kowloon, Hong Kong; 5 Department of Healthcare and Public Health, Korea National Rehabilitation Center, Rehabilitation Research Institute, Seoul, 01022 South Korea; 6 Department of Sport, Physical Education and Health, The Hong Kong Baptist University, Kowloon, Hong Kong; 7 School of Chinese Medicine, The University of Hong Kong, Pok Fu Lam, Hong Kong; 8 School and Graduate Institute of Physical Therapy, College of Medicine, National Taiwan University, Taipei City, Taiwan

**Keywords:** Martial art, Proprioception, Muscle strength, Muscle contraction speed, Older adults, Exercise

## Abstract

**Background:**

To explore the effects of Ving Tsun (VT) sticking-hand training on knee joint proprioception and leg muscular performance among community-dwelling middle-aged and older adults.

**Methods:**

Thirty-three middle-aged and older adults were randomly assigned to either the VT or control group. The VT group received sticking-hand training twice/week for 3 months. Data were collected before and after the intervention period. The primary outcome was knee joint repositioning error, which was measured using knee joint active repositioning tests. The secondary outcomes included the maximum muscle strength and time to maximum muscle strength of the major leg muscles.

**Results:**

No significant group, time, or group-by-time interaction effects were found for the knee joint repositioning error (*p* > 0.05). The maximum muscle strength of the knee flexors increased over time in the VT group only (*p* = 0.038). In addition, the time to maximum muscle strength in the hip extensors and flexors increased over time in both groups (*p* < 0.05). For the knee extensors and flexors, the time to maximum muscle strength increased in the control (*p* = 0.027) and VT (*p* = 0.019) groups, respectively, over time.

**Conclusions:**

VT sticking-hand training could improve the maximum muscle strength of the knee flexors but could not attenuate the age-related deterioration in leg muscle contraction speed nor improve knee joint proprioception among community-dwelling middle-aged and older adults.

**Trial registration:**

ClinicalTrials.gov NCT03318289. Registered on 23 October 2017.

## Background

Falls are a major cause of morbidity and mortality among older adults worldwide. Poor postural control (body balance) is one of the strongest risk factors for falls in the aging population [1]. Postural control involves integrating somatosensory (i.e., proprioception and cutaneous sensitivity), visual, and vestibular inputs in the central nervous system and then generating motor outputs via the musculoskeletal system (e.g., contraction of the leg muscles to generate adequate force) [2]. A previous study suggested that deterioration of lower limb joint proprioception and muscle strength are important contributors to poor postural control among older adults [3]. Therefore, the importance of both joint proprioceptive training (sensory training) and muscle strengthening exercises (motor training) should be emphasized in therapeutic exercise interventions for the middle-aged and elderly to improve their postural control and prevent falls.

The currently used exercise interventions for improving the physical status and balance and reducing the risk of falls among older adults range from flexibility exercises to dynamic balance training. These interventions are usually implemented in clinical and rehabilitative settings [1]. In Hong Kong, tai chi (TC) is a popular fall prevention exercise practiced by older Chinese adults. TC, a soft-style Chinese martial art, has been reported to improve lower limb joint proprioception, muscle strength, and postural control among older adults [4, 5]. However, the traditional forms of TC may be too tedious and complicated to master because they involve a long series of slow, continuous, and predetermined movements [6, 7]. Practitioners may thus drop out from TC programs or be demotivated to continue their training.

In recent years, our research team has tried to explore a more dynamic, interesting, and interactive training method to improve physical health, including lower limb joint proprioception, muscular performance, and postural control, among middle-aged and older adults [8–14]. Regular participation in an exercise program is important for community-dwelling middle-aged and older adults to be able to maintain a healthy, active lifestyle and prevent falls [1, 15]. The authors found that Ving Tsun (VT, also known as Wing Chun), a soft- and hard-style Chinese martial art, may improve postural control [9, 10, 12] and leg muscle strength [10] among community-dwelling middle-aged and older adults in Hong Kong. A research team led by Rostami in Iran [16] also reported that VT practitioners demonstrated lower attentional involvement in postural control when performing the VT basic stance. These reports collectively suggest that VT could be an ideal exercise for middle-aged and older people to improve their postural control and the associated neuromuscular performances. In addition, practicing VT is fun because it involves a lot of interactive, dance-like movements that are practiced with a partner (known as VT sticking-hand training). It is well accepted by middle-aged and older adults; moreover, as demonstrated in our previous studies, it could be a habitual exercise regimen for middle-aged and older adults [8–14].

Therefore, as a continuation of our previous study, in the current manuscript, we aimed to explore the effects of VT sticking-hand training on lower limb joint proprioception and muscular performance among middle-aged and older adults. We were particularly interested in examining the knee joint proprioception of the participants because VT practitioners have many opportunities to position their knees in space when performing different kinds of footwork [17]. Repeated practice of VT footwork might improve selective attention to sensory cues, and thus, neural plasticity might occur in the primary sensory cortex [18, 19]. Training in VT footwork may also improve motor coordination and muscle power generation capacity in middle-aged adults. In fact, a previous exploratory study revealed that the time required to reach peak force in the knee flexor muscles was shorter in the VT practitioners than in the control participants [12]. So, we hypothesized that this novel exercise regimen would help to improve knee joint proprioception, maximum muscle strength, and time to maximum muscle strength (a quasi-measure of muscle contraction speed) of the major lower limb muscles among community-dwelling middle-aged and older adults.

## Methods

### Study design

This single-blinded, randomized controlled trial involved two parallel groups and was registered on ClinicalTrials.gov in October 2017 (clinical identifier: NCT03318289). The Human Research Ethics Committee of the University of Hong Kong approved the study (approval number: EA1602061). Written informed consent was obtained from each participant prior to data collection. All procedures were conducted in accordance with the Declaration of Helsinki.

### Participants

Middle-aged and older adults were recruited from the Un Chau Neighborhood Elderly Center of the Hong Kong Christian Service (HKCS) through notice advertisements. The inclusion criteria were as follows: (1) age > 55 years, (2) ability to move independently, and (3) ability to follow instructions and communicate with others. The exclusion criteria were as follows: (1) unstable medical conditions such as uncontrolled hypertension or diabetes mellitus, (2) a recent injury that could affect test performance, (3) a history of fragility fractures or osteoporosis/osteopenia, (4) significant musculoskeletal disorders such as severe knee osteoarthritis, (5) sensorimotor or neurological disorders, (6) cardiopulmonary disorders, (7) cognitive disorders, (8) regular participation in sports or training in martial arts such as TC, and (9) frailness preventing participation in the VT program.

### Randomization, allocation concealment, and assessor blinding

The middle-aged and older adults were screened by three experienced physiotherapists. Eligible participants were randomly assigned to either the VT or control group. The randomization process involved the use of sealed, opaque envelopes prepared by an independent person to ensure concealed allocation. The first author performed concealed allocation, including the opening of the envelopes. A social worker from the HKCS, who was not a study participant, organized the VT training sessions. The assessors included physiotherapists and research assistants who were blinded to the group allocation; they assessed the included participants before and after the intervention period. The participants were reminded not to disclose their group allocation information to the assessors to ensure assessor blinding.

### Intervention

#### VT group

The participants in the VT group underwent supervised VT sticking-hand training at an indoor sports center two times a week (1 h/session) for 3 months. The VT sticking-hand training protocol (Table 1) was modified from the traditional Wong Sheung Leung method VT drills and designed by the corresponding author, who is a physiotherapist and a senior VT coach. The training protocol was designed to improve the sensorimotor performance and dynamic body balance of the participants. The training program comprises nine offensive and defensive VT sticking-hand drills that are practiced with a partner. Both partners alternate between the roles of attacker and defender. The detailed training method can be viewed at https://youtu.be/ssaYXNGm7hM and can also be found in our previous publications [8, 14]. All VT training sessions were conducted by a VT Athletic Association-certified coach and assistant coach. Feedback regarding the knowledge of the results and performance was provided to each participant to help them master the VT techniques progressively [20]. To ensure safety, the coach-to-participant ratio was approximately 1:8.

**Table 1:** The Ving Tsun sticking-hand training protocol

**Ving Tsun sticking-hand drills** (a video demonstration: https://youtu.be/ssaYXNGm7hM)	**Training frequency**	**Training intensity**	**Training duration**
Warm-up (jogging and stretching exercises)	2/week	20 reps	5–10 min
1. Single sticking-hand exercise in static stance	2/week	20 reps	5 min
2. Double sticking-hand exercise in static stance	2/week	20 reps	5 min
3. Advancing footwork with Taan Sau and retreating footwork with Fook Sau	2/week	20 reps	5 min
4. Advancing and retreating footwork with vertical punches	2/week	20 reps	5 min
5. Pivoting footwork with Taan Sau and vertical punch in response to an incoming punch	2/week	20 reps	5 min
6. Pivoting footwork with Gang Sau to deflect an incoming punch	2/week	20 reps	5 min
7. Advancing footwork with palm strikes in response to an incoming punch	2/week	20 reps	5 min
8. Advancing footwork with shoulder strike in response to a Lap Sau	2/week	20 reps	5 min
9. Advancing footwork with Bong Sau in response to a Lap Sau	2/week	20 reps	5 min
Cool down (jogging and stretching exercises)	2/week	20 reps	5–10 min

All sticking-hand drills are practiced with a partner

#### Control group

The participants allocated to the control group received no VT intervention but continued their daily activities and medical care, if necessary. They were asked not to participate in any martial arts training during the study period.

### Outcome measurements

**Fig. 1:**
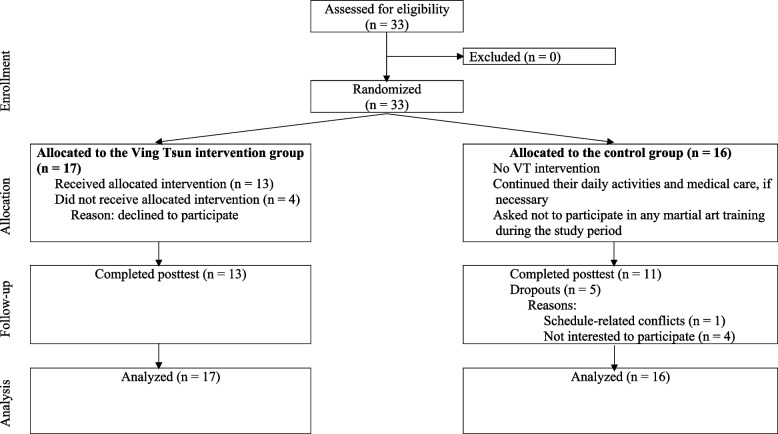
Flow chart depicting the phases of the study

The participants were assessed at baseline (pretest) and shortly after the 3-month intervention period (posttest) at the Un Chau Neighborhood Elderly Center. Figure 1 presents a flowchart depicting the various phases of the study. All participants underwent the following measurements in random order.

#### Primary outcome measure

## Knee joint repositioning error

Knee joint proprioception was measured using an active knee joint angle repositioning test, as described by Fong and Ng [18]. The participants were asked to lay on their nondominant side on a mat and close their eyes during the test. The weight of the participant’s dominant leg was supported by the assessor, with the hip joint angle maintained at approximately 45° flexion. A universal goniometer was attached to the lateral side of the knee joint of the dominant leg to measure the knee joint range of motion under flexion and extension. During the test, the participant’s knee joint was first positioned at 35° flexion by the assessor. Then, the knee was randomly moved by the assessor to a new joint angle between 20 and 75° flexion and maintained in that position for 3 s. The participant was asked to remember this specific knee joint angle, and the angle was recorded. Next, the knee was repositioned to the starting joint angle by the assessor. Five seconds later, the participant was asked to actively position the knee back to the previous joint angle. The angle reproduced by the participant was recorded. Two testing trials were conducted; a 30-s rest period was allowed between the trials. The absolute error, defined as the difference between the original and repositioned knee joint angles, was calculated for each trial. The mean absolute error (in degrees) of the two trials was used for analysis. The smaller the absolute error, the better the knee joint proprioception. The test–retest reliability of this measurement is reportedly good (ICC = 0.775) [18].

### Secondary outcome measures

## Maximum muscle strength and time to maximum muscle strength in the lower limb muscles

The maximum muscle strength and time to maximum muscle strength of the participants’ nondominant hip extensors, hip flexors, knee extensors, and knee flexors were measured using the Lafayette Hand Held Dynamometer (Lafayette Instrument Company, Lafayette, LA), following standardized manual muscle testing procedures [21] and dynamometer placements [22]. The reliability of this testing method has been found to be perfect (ICC = 0.97–1.00) [23]. The accuracy of the Lafayette Hand Held Dynamometer is ± 0.1 kg, and the sample rate is 25 Hz [22]. During the test, the participant was instructed to contract the respective muscle group as tightly and as fast as possible against the manual resistance of the assessor. The maximum muscle strength was generated for 2 s per muscle group to avoid muscle fatigue on repeated testing [8]. Two testing trials were performed for each muscle group. The order of testing was from proximal to distal muscle groups. The average maximum muscle strength (in kg) and the average time to maximum muscle strength (in seconds) of each muscle group were calculated and used for further analysis.

The maximum muscle strength (and endurance) of the ankle plantar flexors was also quantified using a single-leg standing heel raise test. This test is commonly used in clinical settings and has demonstrated high intrarater reliability among adults (ICC = 0.89) [24]. During the test, the participants were made to stand barefoot on their nondominant legs and were asked to raise their heels as high as possible, repetitively. During the process, the participants were required to keep their trunks upright and look forward. An examiner provided finger-touch support to assist with body balance and counted the total number of heel rises. The number of repetitions of heel raises until exhaustion (i.e., no further repetitions could be performed), indicating the maximum strength of the ankle plantar flexors [24], was noted and used for further analysis.

### Statistical analysis

G*Power 3.1.9.2 (Franz Faul, Universitat Kiel, Germany) was used to calculate the sample size. Assuming an effect size of 1.35 (according to Fong et al. [10]), a two-tailed alpha level of 5%, and a statistical power of 80%, 10 participants per group were deemed necessary. Anticipating a 25% dropout rate [13], the requirement for a minimum of 13 participants per group was determined, thus indicating that 26 participants were needed in total.

SPSS version 27 (IBM, Armonk, NY) was used for data analysis. Missing data were handled by applying the intention-to-treat principle (last observation carried forward). Descriptive statistics were used to describe all variables, and data normality was confirmed using histograms. The between-group differences in the demographic characteristics and outcome variables at baseline were compared using the independent *t*-test and chi-square test, as appropriate. Two-way repeated measures analysis of variance (ANOVA, group × time) was used to compare each outcome variable between and within groups. Post hoc analyses were then performed using independent and/or paired *t*-tests, as appropriate. The level of significance was set at 5% (two-tailed).

## Results

From November to December 2017, 33 community-dwelling middle-aged and older adults were recruited and screened for eligibility; all of them were deemed eligible to participate in the study and were randomly assigned to either of the following two groups: VT group (*n* = 17) and no-training control group (*n* = 16) (Fig. 1). The demographic characteristics and outcome variables at baseline were similar between the groups (all *p* > 0.05) (Table 2). Four and five participants dropped out of the VT and control groups, respectively. Their reasons for dropping out and the time at which they did so are detailed in Fig. 1. No significant differences were noted between the baseline characteristics of the participants who dropped out and those who did not. The overall attendance rate of the VT group was 77%, and the total VT training time was 19 h for each participant. All participants were able to continue their usual medical care during the study period and reported no changes in their medication or levels of physical activity. Moreover, no adverse events were reported orally by the participants at the end of the intervention period, indicating that VT sticking-hand drills are safe and suitable for middle-aged and older adults.

**Table 2:** Demographic characteristics of the participants

	**Ving Tsun group (*n* = 17)**	**Control group (*n* = 16)**	***p* value**
Age, years	67.5 ± 6.3	72.1 ± 10.3	0.129
Sex (male/female), *n*	2/15	3/13	0.576
Body weight, kg	55.5 ± 8.9	55.2 ± 7.8	0.908
Height, cm	154.6 ± 7.6	153.5 ± 6.5	0.670
Body mass index, kg/m^2^	23.3 ± 3.5	23.4 ± 3.0	0.889
Physical activity level, metabolic equivalent hour/week	14.4 ± 19.0	14.2 ± 13.0	0.976

Means ± standard deviations are presented, unless otherwise specified

The two-way repeated measures ANOVA revealed no significant group, time, or group-by-time interaction effects for the primary outcome of knee joint repositioning error (all *p* > 0.05). With respect to the secondary outcomes, the time effect was significant for the maximum muscle strength of knee flexors (*p* = 0.009) and ankle plantar flexors (*p* = 0.048). The time effect was also significant for the time to maximum muscle strength in hip extensors (*p* = 0.001), hip flexors (*p* < 0.001), knee extensors (*p* = 0.012), and knee flexors (*p* = 0.003) (Table 3). No significant group, time, or group-by-time interaction effects were found for the other secondary outcomes (all *p* > 0.05) (Table 3).

**Table 3:** Between- and within-group comparisons of outcome measurements

	**Ving Tsun group (*n* = 17)**		**Control group (*n* = 16)**		***p* value**		
	**Pretest**	**Posttest**	**Pretest**	**Posttest**	**Group**	**Time**	**Group × time**
**Primary outcome**							
Knee joint repositioning error, degrees	6.5 ± 8.0	4.6 ± 4.6	4.8 ± 5.7	4.4 ± 4.3	0.552	0.511	0.689
**Secondary outcomes**							
Maximum muscle strength							
Hip extensors, kg	10.8 ± 4.8	11.1 ± 2.9	9.6 ± 3.4	10.5 ± 3.3	0.466	0.466	0.732
Hip flexors, kg	11.0 ± 2.7	11.8 ± 3.7	9.2 ± 2.0	11.7 ± 4.8	0.402	0.083	0.343
Knee extensors, kg	11.1 ± 4.2	10.4 ± 3.9	9.3 ± 1.4	9.9 ± 2.3	0.288	0.980	0.477
Knee flexors, kg	9.3 ± 2.8	10.9 ± 2.8^a^	8.6 ± 3.8	10.2 ± 2.2	0.488	0.009*	0.972
Ankle plantar flexors, single-leg standing heel raise repetitions	12.9 ± 11.6	18.6 ± 5.4	12.8 ± 17.3	19.1 ± 3.0	0.947	0.048*	0.931
Time to maximum muscle strength							
Hip extensors, s	1.7 ± 1.0	2.5 ± 0.5^a^	1.3 ± 0.9	2.3 ± 0.8^a^	0.163	0.001*	0.669
Hip flexors, s	1.6 ± 0.8	2.2 ± 0.7^a^	1.4 ± 0.9	2.5 ± 0.5^a^	0.635	< 0.001*	0.211
Knee extensors, s	1.7 ± 1.0	2.1 ± 0.9	1.6 ± 0.9	2.4 ± 0.6^a^	0.850	0.012*	0.376
Knee flexors, s	1.5 ± 0.9	2.2 ± 0.6^a^	1.7 ± 0.9	2.4 ± 0.8	0.508	0.003*	0.927

Means ± standard deviations are presented, unless otherwise specified

^*^p < 0.05

^a^Within-group changes: *p* < 0.05 compared with the pretest value

The post hoc analyses showed that for the VT group, the posttest value of the maximum muscle strength of knee flexors was increased compared with the pretest value (*p* = 0.038). However, in the VT group, the increase in the maximum muscle strength of the ankle plantar flexors over time was not statistically significant (*p* = 0.089). In both groups, the posttest values for the time to maximum muscle strength in both hip extensors and hip flexors were increased compared with the pretest values (all *p* < 0.05). Moreover, for knee extensors and flexors, the time to maximum muscle strength was increased in the control group (*p* = 0.027) and VT group (*p* = 0.019), respectively, over time (Table 3).

## Discussion

To the best of our knowledge, this was the first experimental study to explore the effects of VT sticking-hand training on lower limb sensorimotor performance among community-dwelling middle-aged and older adults. Three major inferences can be made from this randomized controlled trial: (1) VT sticking-hand training can improve the lower limb maximum muscle strength, particularly the maximum muscle strength of knee flexors, among community-dwelling middle-aged and older adults. (2) However, our VT sticking-hand training program could not shorten the time to maximum muscle strength in the lower limb major muscles of middle-aged and older adults. We found that after the intervention, both the VT and control groups required more time to achieve maximum muscle strength in the major lower limb muscles. (3) In addition, VT training did not improve knee joint proprioception among middle-aged and older adults.

Finding 1 concurred with our hypothesis and partially corroborated the finding of previous studies that have reported that aged VT practitioners have greater knee flexor and extensor muscle strength than their counterparts [10, 12]. An improvement in the knee flexor muscle strength may be related to the special VT sticking-hand training method. Because dynamic footwork, which is usually performed in the half-squat pose, was emphasized during VT sticking-hand training, co-contraction of the knee extensor and flexor muscles was needed to maintain postural stability. This might be an alternative way to strengthen the knee flexor muscles, which are usually undertrained in middle-aged and older individuals [17], without performing specific resistance training exercises. Further research is needed to understand the electromyographic activities of the knee muscles during VT sticking-hand training to confirm this postulation.

In the current study, we used a handheld dynamometer to assess the isometric muscular performance of middle-aged and older adults and found that VT could not improve the maximum muscle strength of the hip extensors, hip flexors, knee extensors, and ankle plantar flexors. These findings were in disagreement with those of our previous studies [10, 12]. In 2017, using an isokinetic dynamometer at 60°/s, we found that compared with the controls, VT practitioners had a higher body weight-adjusted peak torque of the knee extensors and knee flexors [12]. In 2014, we used the five times sit-to-stand test and found that compared with the controls, VT practitioners had greater leg muscle strength [10]. Thus, we postulated that the isometric muscular performances measured in this study do not reflect the actual muscle strength gained through dynamic VT sticking-hand training.

Finding 2 also differed from the findings of our previous study, which showed that the time to maximum muscle strength in the knee flexor muscles was shorter in the VT group than in the control group [12]. This mismatch may be attributable to two reasons. First, our previous study used a cross-sectional design [12], indicating that the faster contraction speed of the knee flexors among VT practitioners than among controls could have been caused by the VT training per se or by other reasons such as genetics. The current study is a randomized controlled trial and confirms that 3 months of VT sticking-hand training could not shorten the time to maximum muscle strength in the major lower limb muscles. Second, the VT practitioners included in our previous study had 10 years of VT experience on average [12]; in contrast, the participants in the current study did not have any experience in VT and received only 3 months of training (VT group). Future randomized controlled trials should aim to increase the VT group’s training duration to confirm these findings. Future studies may also examine the lower limb muscle power or rate of force development instead of time to maximum muscle strength to reflect muscle explosivity.

Contrary to our hypothesis, we found that VT training could not improve knee joint proprioception among middle-aged and older adults [finding 3]. Unlike TC, VT sticking-hand training does not involve any slow bodily movements or require practitioners to pay attention to and remember the position of the limbs in space [25]. Instead, VT training involves relatively fast offensive and defensive drills that challenge the participants’ reactive postural controls. This fast and dynamic nature of VT training may explain why it could not improve static knee joint proprioception. Further studies should examine knee joint kinesthesia and the associated dynamic (reactive) balance performance instead.

This study has three more limitations. First, the participants were not blinded to the group allocations owing to the nature of the VT exercise intervention. This may have introduced some bias in the results. Second, adverse events were reported orally by the participants, and we did not have a formal method of collecting adverse event data. Finally, the current study involved healthy middle-aged and older adults belonging to the Chinese community; thus, the study findings may not be generalizable to Chinese middle-aged and older adults having a disability or to Western populations.

## Conclusions

VT sticking-hand training could improve the maximum muscle strength of knee flexors but could not attenuate the age-related deterioration of lower limb muscle contraction speed or improve knee joint proprioception among middle-aged and older individuals. Clinicians, physiotherapists, and sports coaches should include VT drills in muscle strengthening or fall prevention exercise programs meant for community-dwelling middle-aged and older adults, thereby reducing the injurious fall-related healthcare cost. Community and elderly centers could also provide VT training classes for community-dwelling middle-aged and older adults to improve the health and well-being of the aging society.

### Trial status

The study was registered on ClinicalTrials.gov on 23 October 2017 (clinical identifier: NCT03318289) and completed.

## Data Availability

The datasets used and/or analyzed during the current study are available from the corresponding author upon reasonable request.

## Abbreviations

ANOVA: Analysis of variance
HKCS: Hong Kong Christian Service
TC: Tai Chi
VT: Ving Tsun
